# Implantation of In Situ Gelling Systems for the Delivery of Chemotherapeutic Agents

**DOI:** 10.3390/gels10010044

**Published:** 2024-01-05

**Authors:** Elena O. Bakhrushina, Iosif B. Mikhel, Liliya M. Buraya, Egor D. Moiseev, Irina M. Zubareva, Anastasia V. Belyatskaya, Grigory Y. Evzikov, Alexey P. Bondarenko, Ivan I. Krasnyuk, Ivan I. Krasnyuk

**Affiliations:** 1 Department of Pharmaceutical Technology, A.P. Nelyubin Institute of Pharmacy, I.M. Sechenov First Moscow State Medical University (Sechenov University), Moscow 119048, Russia; bakhrushina_e_o@staff.sechenov.ru (E.O.B.); motigadzh@mail.ru (L.M.B.); comtghostuk@gmail.com (E.D.M.); kashlikova_i_m@staff.sechenov.ru (I.M.Z.); belyatskaya_a_v@staff.sechenov.ru (A.V.B.); krasnyuk_i_i@staff.sechenov.ru (I.I.K.); 2 Department of Pharmacology, A.P. Nelyubin Institute of Pharmacy, I.M. Sechenov First Moscow State Medical University (Sechenov University), Moscow 119048, Russia; 3 Department of Nervous Diseases and Neurosurgery, N.V. Sklifosovsky Institute of Clinical Medicine, I.M. Sechenov First Moscow State Medical University (Sechenov University), Moscow 119048, Russia; mmaevzikov@mail.ru; 4 Clinical Center VitaMed, Moscow 125319, Russia; dralex9071@gmail.com; 5 Department of Analytical, Physical and Colloidal Chemistry, A.P. Nelyubin Institute of Pharmacy, I.M. Sechenov First Moscow State Medical University (Sechenov University), Moscow 119048, Russia; krasnyuk_i_i_1@staff.sechenov.ru

**Keywords:** tumors, chemotherapy, intratumoral implantation, intratumoral injection, in situ implant, stimuli-sensitive systems

## Abstract

Implantation is a modern method of administering chemotherapeutic agents, with a highly targeted effect and better patient tolerance due to the low frequency of administration. Implants are capable of controlled release, which makes them a viable alternative to infusional chemotherapy, allowing patients to enjoy a better quality of life without the need for prolonged hospitalization. Compared to subcutaneous implantation, intratumoral implantation has a number of significant advantages in terms of targeting and side effects, but this area of chemotherapy is still poorly understood in terms of clinical trials. At the same time, there are more known developments of drugs in the form of implants and injections for intratumoral administration. The disadvantages of classical intratumoral implants are the need for surgical intervention to install the system and the increased risk of tumor rupture noted by some specialists. The new generation of implants are in situ implants—systems formed in the tumor due to a phase transition (sol–gel transition) under the influence of various stimuli. Among this systems some are highly selective for a certain type of malignant neoplasm. Such systems are injected and have all the advantages of intratumoral injections, but due to the phase transition occurring in situ, they form depot forms that allow the long-term release of chemotherapeutic agents.

## 1. Introduction

In recent years, many innovative ways to treat cancer have been actively researched. The most common approach to cancer treatment today is chemotherapy. The mechanisms of action of chemotherapeutic drugs are quite broad. Inhibiting the growth of malignancies, stopping the uncontrolled proliferation of cancer cells, and activating apoptosis are the principal effects of chemotherapeutic drugs [1].

The main advantages of using chemotherapy are a high degree of study, scientifically proven efficacy, a large number of drug combinations and treatment protocols, a relatively low cost of drugs compared to other methods of treatment and prevention, a wide range of use, as well as a large number of different drugs for the treatment of each type of cancer.

The main disadvantage of chemotherapy is its extremely high toxicity to the patient, causing a large number of possible side effects that often lead to irreversible changes in the body and even death [2].

Traditionally, chemotherapeutic drugs are administered parenterally [3,4,5]. This approach is logical in the treatment of aggressive metastatic cancers, as it is necessary to obtain maximum bioavailability from each dose of the drug. To date, intravenous chemotherapy has been the most studied and used in the treatment of malignant neoplasms while causing a wide spectrum of side effects associated with high drug toxicity [2,6,7,8].

Over the past few decades, techniques have been developed to improve patient compliance and adherence. The first example is the replacement of parenteral chemotherapy with oral chemotherapy.

The most obvious advantage of the oral administration of chemotherapeutic drugs is the possibility of reducing the number of hospital visits, decreasing stress levels, and increasing patient mobility in daily life [9,10,11,12]. However, it should be noted that oral treatment is most often used as maintenance therapy for cancer, as the effect of the first passage through the liver is preserved, which reduces the bioavailability of chemotherapy. Another disadvantage of oral chemotherapy is the adherence to the outpatient regimen (taking the drug in the correct dose at the correct time). Non-adherence to the outpatient chemotherapy regimen may lead to the development of undesirable consequences (relapse, disease progression, worsening of the patient’s condition) [9,10,11].

In order to combine the efficacy of the parenteral route of administration and reduce the number of hospital visits, as in the case of oral administration, the development of sustained-release chemotherapeutic agents is necessary. To date, the development of implantable delivery systems capable of reducing the incidence of systemic side effects and prolonging the effect of the administered dose—subcutaneous and intratumoral implants—is being actively pursued.

In contrast to intravenous and oral delivery, intratumoral delivery allows the drug to bypass the systemic circulation and reach the tumor, resulting in fewer systemic side effects. The main advantage of such delivery systems for chemotherapeutic agents is their local effect on the target in the body, the malignant tumor [7,8]. The possibility of molecular therapy also shows outstanding results in cancer treatment by targeting the tumor after entering the bloodstream. Through encoded tumor recognition drug release by interacting with the microenvironment in the tumor, the body bypasses the problem of general toxicity, and the maximum amount of the drug reaches the tumor [13,14]. As with intratumoral administration, subcutaneous implantation has a number of advantages: controlled drug release, the elimination of the need for multiple drug administration, and the maintenance of equilibrium drug concentrations after implantation [15,16,17,18].

These implantation methods become even more attractive when using biodegradable and non-toxic polymers that can form their structure at the site of injection (in situ). By using different types of in situ polymers, it is possible to regulate the release rate of the antitumor drug [8,19].

This lack of interest may be related to the small number of published reports of clinical trials on the use of such formulations, i.e., intratumoral implants. At the same time, the literature search revealed a sufficient number of studies, including developmental and in vivo studies in animal models, demonstrating the efficacy of intratumoral therapy.

The aim of this review is to systematize modern approaches to intratumoral therapy and to justify its relevance as an alternative to the widely used subcutaneous implantation and intratumoral injection of chemotherapeutic agents.

## 2. Subcutaneous Implantation

Subcutaneous implantation entails introducing a structure comprised of a distinct biopolymer containing an active pharmaceutical ingredient (API), which facilitates optimal drug release and targets the intended location. This therapy option is considered an effective alternative to conservative antitumor treatment, which is known for poor oral bioavailability and high toxicity [20]. Since the development of the first drug-coated subcutaneous implant in the 1930s, investigators have achieved tremendous success in prolonging and targeting API action using biomedical implants [21]. There are two types of implantable systems: reservoir-type and matrix-type. In reservoir-type implants, the drug core is covered by a semi-permeable polymer membrane that controls the drug release, which is dependent on the rate of fluid circulation in the implant system. But, reservoir-type implants are often not recommended because of the likelihood of a high dose being released at a time. Matrix-type implants contain evenly distributed APIs in a polymer matrix [22]. They provide the desired dual-phase drug release by passive diffusion. The first phase consists of a burst followed by a slow controlled release that maintains a therapeutic drug concentration [17].

Thus, Pengxiao Cao et al. [23] developed polymeric subcutaneous implants (millirods, which are narrow cylinders of red color) made of ε-caprolactone. These millirods were tested for the local delivery of green tea polyphenols, both in vitro and in vivo, in cancer treatment. Green tea polyphenols were released from the implants continuously over time, and the drug load was the main determinant of release completeness. The in vitro and in vivo (rat model) release rate followed similar kinetics for 16 months. The active ingredient was released from the implants by a simple diffusion process. The initial burst release of the drug embedded in the implants could be optimized by coating the implants with empty polymers and adjusting the drug load. These implants could be used locally at the tumor site as an alternative strategy.

Amirhosein Kefayat et al. [16] investigated the possibility of forming cylindrical subcutaneous implants based on chitosan nanoparticles loaded with mebenadzole and folic acid for the treatment of mammary tumors. The implants completely degraded in the mouse body 15 days after implantation and caused an over 50% reduction in average tumor volume. Moreover, the implants were completely biocompatible based on histopathological and blood biochemical analyses. Thus, these biodegradable and biocompatible implants may be a suitable choice for further breast cancer treatment experiments.

The first original implant approved by the FDA was Zoladex^®^ (API—goserelin) for a 14-week treatment of prostate and breast cancer. The drug is a one-centimeter-long PLGA-based implant with a sustained release of API that is inserted into the anterior abdominal wall using a special applicator syringe. When released, goserelin reduces estrogen levels produced by the ovaries [24].

The registered drug Lupron Depot^®^ (API—leuprorelin) is composed of PLGA microspheres loaded with a pharmaceutical substance for prostate cancer treatment. After subcutaneous or intramuscular injection, the microspheres are immobilized at the injection site. Drug molecules are released from the immobilized spheres and diffuse locally into tissues, then enter the vasculature, which distributes the drug throughout the body, inhibiting gonadotropin secretion and exerting antitumor effects [25].

A similar dosage form is presented in another registered drug, Decapeptyl^®^ (active substance triptorelin), which, like the previous example, is PLGA microspheres loaded with a drug substance for prolonged action and treatment of uterine myoma and hormone-dependent prostate carcinoma. It is also administered by subcutaneous and intramuscular injection [26].

In addition to traditional chemotherapeutic drugs, subcutaneous implants are offered to create antigen-specific antitumor immunity for long-term tumor elimination through long-term immunomodulation. A reservoir that can be refilled according to the patients’ needs can be used as a platform.

Dixita Ishani Viswanath et al. [27] developed an implantable subcutaneous device for antigen-specific antitumor immunomodulation, “NanoLymph”, based on autologous dendritic cells. “NanoLymph” consists of a platform with two reservoirs for the prolonged release of immunostimulants through a nanoporous membrane and hydrogel-encapsulated antigens for the local activation of immune cells. The biocompatibility of NanoLymph was evaluated in mice and proved that the drug was non-cytotoxic and biocompatible, as the mice remained healthy with no signs of abscess in the implantation area. It was also observed that the drug showed a constant and sustained release, thus achieving therapeutic effects.

Another example could be the study of Sidi A. Bencherif et al. [28], where they were involved in the fabrication of injection sponges by cryogelation. The cryogels were made of alginate encapsulated with granulocyte-macrophage colony-stimulating factor (GM-CSF), which acts as a dendritic cell (DC) enhancement factor, and cytosine-phosphodiester-guanine oligodeoxynucleotide (CpG ODN), which serves as a DC activator. So, unlike traditional nanoporous hydrogels, alginate cryogels are elastic, soft, spongy materials that can withstand large deformations and can be easily passed through a syringe [29]. Based on the results, these cryogels provide the sustained release of immunomodulators and may serve as a platform for the vaccination of cancer cells.

However, despite its simplicity, convenience, and higher efficacy compared to the systemic administration of the drug, it is logical to assume that subcutaneous implantation may not have the same bioavailability and targeting as intratumoral implantation, which involves the direct injection of the drug into the tumor. For this reason, we are going to take a closer look at intratumoral implantation, which is of more interest to us, and we are going to look at all of its aspects, innovations, and drawbacks.

## 3. Intratumoral Implantation

### 3.1. Fundamentals of the Intratumoral Implantation Method

Most currently known therapies for cancer involve the introduction of highly toxic drugs into the systemic bloodstream. As a result, many serious side effects occur, highlighting the need to develop treatment modalities that target a specific area or organ, such as intratumoral implants [30].

In the case of intratumoral implantation, the implant with the active substance is inserted directly into the malignant tumor in order to reduce or destroy it. It is also possible to insert the implant into the area around the tumor (peritumoral implantation). Currently, the most common active substances used in intratumoral implantation are chemotherapeutic drugs.

As previously discussed, chemotherapy is a widely used approach for cancer treatment, although it has drawbacks. High doses of chemotherapy can cause systemic toxicity, and the presence of large tumors can lead to poor pharmacokinetics due to interstitial fluid pressure, hindering transcapillary transport and preventing substance penetration into the tumor [31].

Local chemotherapy using drug delivery systems can provide continuous drug release and high drug concentration at the tumor site that results in minimal systemic toxicity compared to conventional chemotherapy. Additionally, it is effective at preventing the local recurrence of post-resection cancer [32,33]. Intratumoral chemotherapy has also been proposed as a way of sensitizing the tumor prior to systemic chemotherapy and radiation therapy [34].

The potential of intratumoral implantation as a method of administering substances with high anticancer properties but limited bioavailability is a compelling area of study [35,36]. GLIADEL^®^ Wafer is a biodegradable implant that received approval for the treatment of malignant brain tumors, including glioblastoma and glioma, in 1996. Carmustine, a cytostatic drug and the active ingredient in this implant is released from the matrix of the implant, which is inserted in the space left after surgery. As a result, the patient receives high-dose local chemotherapy after the tumor removal for a period of time. The implant is a white or pale-yellow plate with a diameter of approximately 1.45 cm. It contains 7.7 mg of carmustine and 3 mg of polyanhydride copolymer [37].

The post-surgery use of GLIADEL^®^ Wafer for glioblastomas substantially improved the efficacy of the procedures performed, providing optimism for both patients and oncologists [38].

Dan Castro et al. [39] evaluated the effectiveness and safety of an intratumoral injectable gel containing cisplatin and adrenaline for managing local control and symptom relief in patients with squamous cell carcinoma of the head and neck that is recurrent or metastatic. In a double-blind phase III study, eighty-seven patients were assigned randomly to either the cisplatin/adrenaline intratumoral injection gel or placebo group. Most participants who were administered cisplatin/adrenaline gel obtained a palliative impact in comparison to placebo-treated individuals. Renal and hematologic toxicities were infrequently detected. The side effects observed included local pain and skin irritation, which resolved spontaneously without complications.

Pan et al. [40] used a PLGA-PEG-PLGA polymer gel matrix and supplemented it with trastuzumab, a breast cancer treatment drug, and collagenase to create a temperature-sensitive delayed-release hydrogel. Through peripheral injection into mouse tumors, the hydrogel systems demonstrated minimal toxicity and were able to persist sustainably in the peritumoral region for over 20 days. The treatment decreased the collagen density and increased apoptotic cell death in tumor tissue. As a result, it provided better treatment with improved effectiveness and decreased toxicity as compared to other control groups in mice. Additionally, a quarter dose of the prolonged-release gel system exhibited better efficacy after a single release as compared to administering it intravenously four times.

Intratumoral implants differ from intratumoral injections primarily in that the former remain at the site of application for a longer period of time, resulting in less toxicity in the body. If implants are being considered for drug delivery, the biodegradability of the implant matrix must be considered in order to allow for later removal from the body without invasion and to address the risk of inflammation or unanticipated infection [41,42].

### 3.2. Potential Risks of Intratumoral Implantation

Despite the excellent results achieved and predicted for intratumoral and subcutaneous management, there are still reasons why, to date, this technique does not have widespread application and still requires careful study.

For instance, it is essential to consider the tumor microenvironment, which is characterized by high intratumor pressure. Although highly permeable vessels and lymphatic dysfunction [43] can aid in targeting and delivering drug particles, the elevated pressure poses a risk of poor penetration of the drugs deep into the tumor [44]. During intratumoral administration, it is important to consider the implant density and its diffusion abilities. Unstable and disorganized tumors with low diffusion can cause uneven distribution or even the excretion of the drug without retention in the tumor.

It should be noted that the capacity and the depth of penetration of the intratumoral tool for implant delivery are limited [45]. This challenge may be due to deep solid tumors located behind healthy tissues, posing a risk of the needle striking them, whereas surgical intervention may be highly invasive and unsuitable. This problem is less significant with conveniently accessible cancer tumors. For instance, a study conducted by T. Peretz et al. [46] revealed that the common complications of implant treatment for pancreatic carcinoma were gastrointestinal bleeding, fistulas, and intra-abdominal infections.

From the previously mentioned drawbacks, the issue of dose control difficulty arises. The external microenvironment of the tumor, along with tumor interstitial fluid pressure, low diffusivity, and instability due to highly permeable vessels, may result in unintended consequences like inadequate drug amounts at a low administration rate due to the intratumor environment. In contrast, administering a high dose and high rate of drugs for a disproportionately small tumor can result in excessive drug leakage and cause off-target toxicity. Intratumor administration requires a much lower dose than systemic administration, given the difference in tumor accumulation efficiency. Therefore, the dose and administration rate should be specifically controlled based on the size of the tumor tissues [47].

In addition, the immune response of the body to the implanted biomaterial must be taken into account [48]. Concerns arise about acute and chronic inflammation, the formation of giant foreign body cells, and the formation of a fibrous capsule. These issues pose significant challenges to the usage of subcutaneous and intratumoral implants and pose potential threats to the patient’s health and safety.

Despite the presence of drawbacks, these can potentially be resolved in the future. For instance, the issue of uneven drug diffusion and unpredictable dosage within tumors can be resolved through the use of multi-needle injections. This method ensures the even distribution of injections from all angles, thereby preventing drug withdrawal and increasing efficacy [47]. The issue of instrument penetration into the tumor without harming surrounding healthy tissues can likely be addressed by utilizing laparoscopy as an alternative to invasive surgery. Additionally, efforts are underway to address the problem of excessive foreign body reactions through the development of a biomaterials database and the study of interactions between the materials used and host tissues to gain insight into the underlying mechanisms of these reactions [48]. The potential for addressing the current limitations is optimistic, providing validation for the viability of the implantation procedure.

### 3.3. Recent Developments in Intratumoral Implantation

Currently, there are many different studies aimed at creating intratumoral implants with different agents, which can be chemotherapeutical drugs as well as targeted radiotherapeutic agents.

The development of intratumoral implants with various chemotherapeutics is ongoing.

In a study by L. Gao et al., an implant was developed for sarcoma treatment with cisplatin, a cytostatic drug that is one of the most widely used in tumor chemotherapy. The drawbacks of the drug are its high toxicity, broad spectrum of side effects, and low solubility [49]. These disadvantages limit its use but also give rise to a large number of studies to increase the efficacy of cisplatin-based drugs. This study attempted to use the implant in vitro and also in mice with sarcoma tumors. The implant was made by pressing a mixture of cisplatin, polylactic-co-glycolic acid (PLGA), and polyethylene glycol-4000 (PEG-4000). The drug, in the form of a cylindrical implant, was injected locally into the tumor area using a modified trocar. An in vitro study found that 25% of the drug was released in the first 10 h after administration, with the remainder released at a constant rate thereafter and completely released after 200 h. The in vivo study showed that 25.3% of the drug was released on the first day, approximately 53% of the drug was released after 5 days, and the mean cumulative release percentage reached 94% on day 25. In evaluating antitumor efficacy, tumors grew rapidly in mice in the control group, complete tumor regression was observed in 40% of mice in the low-cisplatin (CDDP-L) implant group, and 70% regression was observed in mice whose implants contained a two-fold higher dose of cisplatin (CDDP-L) [50].

Cisplatin was also the subject of a study by B. Yavuz et al. in the treatment of neuroblastoma. In this study, cisplatin also showed fewer side effects in mice compared to systemic administration, suggesting the possibility of increasing the dose of cisplatin for local intratumoral use. The experiment used silk reservoirs made from Bombyx mori cocoons in which cisplatin was placed and then injected into the tumor after incision. This specific method of implantation showed that increasing the dose of cisplatin significantly suppressed tumor growth. For example, when a control reservoir without the drug was injected, a tumor volume of 500 mm^3^ was reached after 1.2 ± 0.3 days; when 0.2 mg of cisplatin was injected, it took 4.2 ± 1.8 days; and when 0.5 mg was injected, the tumor reached the required volume after 17.6 ± 6.4 days [51].

C. Federico et al. conducted a study comparing systemic exposure and exposure by intratumoral implantation on cervical tumors in vivo. This study showed that the efficacy of local treatment was superior to that of systemic therapy. In addition, the implantation of cisplatin with polyethylene glycol-3350 (PEG-3350), polyethylene glycol-400 (PEG-400), and nitric acid increased the sensitivity of tumor cells to radiotherapy [52].

Intratumoral implantation has also been studied as a means of delivering immunotherapy, specifically monoclonal antibodies (anti-CD40 agonists). The drug release in this study was performed by a special system called NDES (nanofluidic drug-eluting seed). NDES is an implantable intratumoral platform for the controlled release of anticancer therapeutics. The device consists of a radiotransparent stainless steel reservoir coated with epoxy resin and a silicon nanofluidic membrane. Intratumor implantation is performed by trocar injection, similar to the clinical delivery of radioactive sources for brachytherapy using special applicators [53,54,55,56].

In the study by P. He et al., an experiment was conducted with an attempt to use an implant made of PLGA and PEG-4000, where doxorubicin, a cytostatic drug with high efficacy in the treatment of cancer, as well as many side effects and low absorption, served as the active ingredient [57]. The implant was made by fusing three components and then injected into a mouse osteosarcoma tumor. The main finding of the study was that the systemic concentration of doxorubicin was much lower than that of systemic administration. The highest concentration was found at the implantation site, confirming once again that intratumoral implantation not only has a more targeted effect on malignant formation but also significantly reduces the overall burden of toxic effects on the body [58].

In the study by C. Wu et al., etoposide was used as the substance for intratumoral implantation. The implant was prepared on the basis of PLLA (50%) and PEG-4000 (40%) and by the direct pressing method. During the experiment, the method of implant preparation was found to be simple and reproducible. The resulting implants were characterized by their easy storage and showed good drug and excipient compatibility. Local chemotherapy using etoposide implants provided a high drug concentration in the target area and avoided excessive drug accumulation in other tissues of the body. In the in vitro study, a 14% drug release was observed in the first 2 h, followed by a 44% drug release within 24 h, and finally, a 92% drug release after 8 days of follow-up. In the in vivo release study, 71% of the drug was not released until the 10th day of observation, and complete release was not achieved until 45 days [31].

Intratumoral implantation has been used to introduce nanoparticle-based radiosensitizers to increase the efficacy of subsequent chemoradiation therapy [59].

In reviewing a number of clinical trial reports, it was found that intratumoral injections are more commonly used as they are more popular than implants. This is mainly due to the ease of manipulation, greater safety, and speed of the study and its conclusions.

In a clinical study by Neil R Sharma et al. [60], patients were treated with microparticles of paclitaxel that provided depot AFI within the tumor. The drug delivery was by endoscopic ultrasound with a fine needle injection. The drug was well-tolerated and induced an immune response. Patients with inoperable locally advanced pancreatic cancer were able to have surgery to remove the tumor in some of the study participants, which is a good outcome.

Patients in the clinical trial by Shi-Yue Li et al. [61] received courses on intratumoral injections of paratoluenesulfonamide, which is used to treat non-small-cell lung cancer. The course was continued until the tumor size decreased by 50% or more. Intratumoral injections of paratoluenesulfonamide were very effective and well-tolerated as a palliative therapy, providing hope for an effective treatment in the near future.

There was also a study by Yasuhiro Shirakawa et al. [62], which aimed to evaluate the efficacy of intratumoral administration of telomelisin for the therapy of esophageal cancer in patients with contraindications to standard chemoradiotherapy. As a result, these multiple courses confirmed their benefit for patients with esophageal cancer ineligible for standard treatment.

Bo Zhang et al. [63] considered genetically engineered oncolytic herpes simplex virus type 2 as a monotherapy for the treatment of solid tumors. The intratumoral administration of the virus was well-tolerated and showed prolonged antitumor activity in patients with metastatic esophageal and rectal cancer, with good tolerance and immune response, which offers interesting prospects for the development of effective monotherapies.

And another promising study was conducted by Jeevinesh Naidu et al. [64], where they evaluated the results of combined chemotherapy and the intratumoral administration of implantable radioactive phosphorus-32 under the control of endoscopic ultrasound in locally advanced pancreatic adenocarcinoma. The success consisted of a reduction in the size of the neoplasm in several patients, leading to successful tumor resection.

It cannot be overlooked that intratumoral injections have been studied much more than intratumoral implants. This may be primarily due to the difficulties in delivery and use of the implant for therapeutic purposes. The disadvantages of intratumoral implantation include internal tumor pressure due to improper lymphatic drainage, which may lead to tumor rupture due to internal compression. It should also be noted that implants can lose their shape during insertion, which can affect the quality of drug release, not to mention the damage that can be caused by an impractical insertion method. Therefore, a promising alternative that combines the qualities of a liquid injection and a solid implant should be considered.

The implants of the next generation are rightly called in situ implants, which are injected locally in a liquid state, and then, under the influence of various factors, they form a gel matrix.

## 4. Intratumoral In Situ Implants

### 4.1. Retrospective and Classification of Intratumor In Situ Implants

The earliest study of in situ intratumoral implants dates back to 2002. American scientists MM Amiji et al. attempted to introduce the cytostatic drug paclitaxel into the melanoma tumor of mice. The polymeric matrix of the implant was based on a block copolymer of polyethylene oxide and polypropylene oxide—Poloxamer 407. The stimulus for the phase transition, in this case, was the temperature inside the tumor. The study showed that the implant was highly effective, which is likely to have stimulated interest in further research in this area. Melanoma tumors in mice were treated with normal saline, a gel containing poloxamer, a saline solution with paclitaxel, and a gel containing poloxamer and paclitaxel. The gel loaded with paclitaxel showed the best results, significantly slowing tumor growth [65].

Currently, there are studies describing different strategies to achieve phase transition within the tumor, which form implants in situ. Experts distinguish physical (temperature, light, electric, and magnetic fields), chemical (pH, redox potential, and solvent composition), and biological (under the influence of enzymes, glucose, etc.) stimuli for phase transition inside the tumor.

Each of the above factors is subject to correction by the proper selection of excipients and adjustment of their concentration [66].

### 4.2. Thermosensitive In Situ Implants

The most commonly used phase transition factor in studies is temperature, which reaches 37 °C in the tumor. At room temperature (~25 °C), the implant is in a liquid state, but when injected into the tumor, it transforms into a gel-like substance that has a prolonged release of the active pharmaceutical ingredient.

For the past twenty years, the method of temperature-induced phase transition has been under active investigation.

To date, poloxamers have been the most studied thermosensitive polymers. Poloxamer 407, at a concentration of 16–30%, exhibits thermosensitive properties that are actively used to create thermoreversible in situ systems [67].

In a study by M. Xu et al. from China [68], Poloxamer 407 was used in combination with Poloxamer 188 and Solutol^®^ HS15 to form a thermoresponsive hydrogel containing nanoparticles. The study demonstrated the antitumor effect in the order of decreasing efficacy: thermoresponsive hydrogel with docetaxel-loaded micelles > docetaxel micelles > docetaxel injection.

G. Brachi et al. also created a nanoparticle-loaded hydrogel using poloxamer 407 to produce a three-dimensional hydrogel matrix for the subsequent treatment of glioblastoma after in vivo testing. The result of the study was that the nanoparticle-loaded hydrogel retained the drug at the injection site 10–12% longer [69].

In a study by H.-K. Liang et al. compared the efficacy of aqueous carboplatin and hydrogel carboplatin (oxidized hyaluronic acid/adipic acid dihydrazide) administration with and without adjuvant radiotherapy in vivo. The results showed a clear advantage for hydrogel implant administration over the intratumoral injection of aqueous solution. The gel formation temperature during the experiment was 37 °C [70].

There were also combinations of Poloxamer 407 with chitosan. For example, MH. Turabee et al. used modified *N*,*N*,*N*,*N*-trimethylchitosan, which has better water solubility over a wide pH range than natural chitosan, mixed with Poloxamer 407 to improve the mechanical properties of the latter, resulting in a heat-sensitive docetaxel gel for the treatment of brain tumors. In an in vitro trial, docetaxel embedded in the polymer combination was significantly more effective than traditional docetaxel. The release of docetaxel from the combination was also shown to be much higher at pH = 7.4 than at pH = 5.5 when measured over 30 days [71].

Modified chitosan has potential applications in the creation of thermosensitive matrices and in combination with other polymers. Combinations of chitosan with b-glycerophosphate have been widely studied in the scientific literature. The successful interaction of the molecules of these two substances and their further influence on the thermosensitivity of the implant was achieved by the fact that chitosan molecules are cationic in nature, while b-glycerophosphate has an anionic nature.

Recent studies with chitosan have used other, mostly inorganic, polymers as the basis for intratumoral implants. For example, effective combinations of chitosan with polycaprolactone [72] and dialdehyde polyethylene glycol [73] have been reported. In the studies reviewed, improved results in terms of antitumor activity and drug release were demonstrated to varying degrees compared to the use of the chitosan/b-glycerophosphate combination alone.

There are also known cases of using chitosan as a mono-matrix formulator to create implants with thermal sensitivity. One of the first studies conducted with chitosan showed that tumor inhibition in mice was 38–40% higher after the administration of the implant containing paclitaxel compared to saline injected in the control experiment. A prolonged and controlled drug release from the matrix was observed [74]. There have also been studies attempting to administer the chitosan matrix with many other pharmaceutical agents, such as camptothecin, which also showed more successful efficacy test results than when camptothecin was administered systemically. It was hypothesized that the efficacy was due to the fact that the created matrix prolonged the effect of the drug on the tumor [75].

The most advanced solutions in the selection of a suitable polymer for creating thermoreversible gels are polyesters such as polylactic acid (PLA), PLGA, or polycaprolactone (PCL).

An example of the formation of a polyester gel is the experiment performed by D. Meng et al., where tamoxifen nanoparticles were mixed with PLGA-PEG-PLGA copolymer solution. In this study, a clear dependence of the phase transition temperature on the copolymer concentration in the solution was found, and a much less significant dependence of the phase transition temperature on the pH of the solution was demonstrated. It was observed that the elimination half-life of tamoxifen as part of the solution injected into the tumor was 13.08 ± 1.14 h, while the elimination half-life of tamoxifen in the polymer matrix was 137.95 ± 11.72 h. Moreover, tamoxifen was detected in tumor tissues 500 h after administration in the gel formulation. The half-life decreased with decreasing pH, i.e., at pH = 7.4, the half-life was 201.91 ± 7.25 h, and at pH = 6.6, it decreased to 188.55 ± 11.03 h [76].

Poly(D,L-lactide) (PDLLA) monomer has also been used to prepare PDLLA-PEG-PDLLA copolymer incorporated into a hydrogel loaded with two drugs, gemcitabine and cisplatin. The composition, despite the presence of two drugs that potentially increase the phase transition temperature of the matrices, showed a stable phase transition at 35 °C, which can be considered a promising direction [77].

A study by HF. Darge et al. [78] used PDLLA as the base for a hydrogel containing bevacizumab and doxorubicin loaded into micelles using the same polymer. As a result, it was demonstrated that intra-tumor administration of bevacizumab and doxorubicin resulted in 57.87 ± 1.22% tumor inhibition, while administration of a formulation consisting of bevacizumab and doxorubicin micelles on a hydrogel base resulted in 80.03 ± 3.07% tumor inhibition.

The work of AM. Al-Abd et al. showed satisfactory results in terms of the phase transition temperature and prolongation of action of the polymer polyorganophosphazene, administered in combination with doxorubicin into the tumor in a biological mouse model. The gelation termination temperature was 34 °C, and the drug was retained in blood and tumor for one month after administration [79].

Increasing the directivity of thermosensitive in situ systems is also possible by locally influencing the area of ultrasound injection. In the study by S. Jeganathan et al. [80], a significant enhancement of doxorubicin release and distribution from a PLGA-based implant containing nanoparticles was demonstrated after short-term ultrasound exposure. Tumor volume was measured 20 days after drug administration and all necessary procedures: in the case of in situ doxorubicin implantation without ultrasound, the tumor volume reached 1832.74 ± 467.83 mm^3^, but when the same implant was administered with additional ultrasound exposure, the tumor reached only 481.20 ± 319.73 mm^3^.

The combination of intratumoral in situ implants with ultrasound may be useful to address some of the problems of local drug delivery in cancer treatment and may serve as an effective non-invasive method to improve the low clinical success rate of local drug delivery systems [81].

### 4.3. pH-Sensitive Intratumoral Implants

In addition to thermoreversive compositions, there is a lot of research and development of pH-sensitive implants. The essence of their function is to respond to an acidic environment in or near the tumor as a result of local pathological acidosis. Such a result is achieved in two ways: by choosing a pH-sensitive polymer with many weakly basic functional groups or by incorporating acid-labile bonds either in the polymer network or between the polymer and the drug [82].

In a study by Z. Xu et al., a pH-dependent polymer, polyethylene glycol-polydiisopropylaminoethyl methacrylate (MPEG-PPDA), capable of forming micelles at very low concentrations, was introduced. In the experiment, a significant increase in doxorubicin release from micelles at pH = 5 was demonstrated at the moment of micelle penetration from tumor tissue (pH = 6.8) into the cell cytoplasm [83].

The polyethylene glycol hyperbranched polyacylhydrazone (PEG-HPAH) used in the study by J. Yu et al. As the pH of the medium decreased, the release rate of docetaxel and doxorubicin increased, reaching a maximum (81% at the 60th hour of the study) at pH = 5 [84].

An example of an experiment with a different strategy to achieve pH sensitivity is a study by J. Liao et al. in which doxorubicin was released from the polymer by hydrazone bonding between it and hyaluronic acid at a pH in the range of 4.5–5.5 [85].

In 2018, J. Xu et al. conducted a study that highlighted the difference in the amount of drug release from the polymer with and without hydrazone bond inserted. The results of the experiment showed that the hydrazone bond has a significant effect on the pH sensitivity of the polymer. Understanding this process allows further studies of pH-sensitive polymers for use in the field of intratumoral implantation [86].

Dextran phosphate has also been shown to be sensitive to pH, as demonstrated by the study of SO. Solomevich et al., where they evaluated the released amount from the implant as the pH decreased from 6.8 to 1.2 [87].

The paper by Wu Y et al. [88] described the development of an in situ intratumor implant based on *n*-butyl-2-cyanoacrylate (NBCA) and ethyl oleate, and the sol–gel phase transition was activated by anions in body fluids or blood. The resulting implant solidified within seconds after contact with body fluids or blood, and the in vivo degradation time of the implant could be monitored. In addition, the pore sizes formed by NBCA polymerization could be reduced by increasing the concentration of NBCA in the implants. Antitumor experiments in animal models showed that the average growth inhibition rate of xenografted human breast cancer cells using an in situ implant loaded with paclitaxel (40% NBCA) was 80%, and tumors in some mice were completely eliminated after a single injection of the drug. For the epirubicin-loaded in situ implant (50% NBCA), the average growth inhibition rate of xenografted human liver cancer cells was 58%.

### 4.4. Phase Sensitive (Phase Inversion) In Situ Implants

There is also some data on the potential of using phase-sensitive in situ implants for intratumoral delivery. These systems show the ability of phase inversion when placed in situ—the bioindifferent solvent diffuses into the surrounding soft tissues or biological fluids, and a solid or visco-plastic implant characterized by modified release is formed at the site of application. The main disadvantage of such systems is the phenomenon of “explosive” release, characterized by a sharp release of the drug from the implant in the first hours after administration. In the work of Narayanan Kasinathan et al. [89], the results of the pharmaceutical development of a QbD standard (ICH Q8) phase-sensitive implant with curcumin nanoparticles as the active ingredient, possessing, among other things, antitumor activity, were presented. This is a phase-sensitive system based on polycaprolactone (PCL) and PLGA that uses acetone as a solvent. In vivo antitumor activity studies in a Swiss albino mouse model showed significant tumor shrinkage, and in vitro studies showed no characteristic burst release for the PLGA-based implant [90].

### 4.5. Photosensitive In Situ Implants

In recent years, hydrogels containing UV-responsive elements have been studied along with other stimuli-responsive compositions.

A study by D. Zhao et al. developed an in situ doxorubicin implant based on the polyethylene glycol-polynitrobenzyl glutamate polymer. The presence of O-nitro groups in the structure of this polymer gives it the properties of photodegradation, more specifically, the irreversible photodegradation of O-nitrobenzyl ether bonds with the help of UV light. The main disadvantages of this method are considered to be the low penetration of light, which makes it possible to treat only tumors located ~1 cm from the skin, as well as the high risk of skin damage. In a 24 h study without UV exposure, ~10% of doxorubicin was released, but when exposed to UV light, approximately 80% was released in 24 h. Increased cytotoxicity was also demonstrated: doxorubicin-loaded micelles reduced tumor cell numbers to 59% and 39% without and with UV light, respectively [91].

Near-infrared (NIR) light can also be used to achieve a photosensitive phase transition. In a study by C. Ruan et al., the phase transition was achieved by intratumoral delivery of cisplatin in a matrix based on *N*-phenylglycine-polyethylene glycol polymer and further laser exposure of the area. In addition to light exposure, the polymer was also exposed to temperature, which also influenced the formation of the implant of the required efficiency and significantly reduced the tumor size [92].

The study by Wei Wu et al. [93] described the use of gemcitabine hydrochloride liposomes for the treatment of osteosarcoma injected intraosseously as part of a photosensitive in situ implant. The implant developed was a photocrosslinkable hydrogel based on gelatin methacryloyl. The in situ formation occurred under the influence of ultraviolet light of 6.9 mW/cm^2^d (360–480 nm) for 10 s. In vitro studies showed that the release of gemcitabine hydrochloride continued for 4 days. In addition, the photocrosslinkable hydrogels showed a good ability to inhibit osteosarcoma in vivo in Balb/c mice bearing MG63 cells.

### 4.6. In Situ Implants Formed by Other Stimuli

Redox reactions are less studied but are rapidly gaining popularity as a way to activate the phase transition. In a 2020 study, Zou Q. et al. demonstrated the ability of an implant based on a reduced diphenylalanine dipeptide derivative to undergo in situ formation within a tumor. In the experiment, almost complete tumor destruction was observed under the influence of the injected polymer in combination with laser treatment [94]. The essence of the method is the incorporation of disulfide bonds into polymers that can be broken by the action of a reducing agent.

Enzymes are the most specific factors that provide maximum selectivity of in situ systems administered within the tumor. Enzyme-selective agents usually target one or more substances released during the pathogenesis of a particular malignancy. Chinese scientists created biodegradable micelles of doxorubicin and PDLLA-PEG-PDLLA, which were then loaded into hyaluronic acid containing a peptide that responds to matrix metalloproteinase-2, which is overexpressed in many cancers. The method developed allowed the drug to interact with cancer cells in a targeted manner [95].

### 4.7. Multi-Stimulation In Situ Implants for Intratumoral Delivery

To date, studies involving implants that respond to two or more factors simultaneously are gaining popularity. One of the first such studies was the YI study by Cho et al. in 2009. In this study, doxorubicin in a composition with a mixture of poloxamer 407 and chito-oligosaccharide, with the help of double exposure to temperature and light, formed an implant that increased the survival rate of mice in in vivo tests up to 100% within 30 days [96].

Another combination of factors was used in a study by S. Khan et al. In this case, the pH and temperature were used to achieve the desired effect. Poly-*N*-vinylcaprolactam sodium alginate polymer is biocompatible, thermosensitive, non-toxic, hydrophilic, and water-soluble at room temperature and, when injected into the body, is exposed to the acidic tumor environment and elevated temperature compared to room temperature, after which it acquires a gel-like consistency and releases the drug for a long time [97].

An example of a multi-sensitive form of drug delivery is the study by G. He et al. in which an implant consisting of gelatin and polydopamine nanoparticles and doxorubicin as an active ingredient was exposed to four factors simultaneously, such as pH, enzymes, light, and temperature. Gluconodeltalactone, added for acidification, affected the polymer, causing the charge reversibility of gelatin nanoparticles. MMP-2/9 enzymes acted on the gelatin nanoparticles to induce the release of doxorubicin. In turn, polydopamine nanoparticles converted NIR light into heat, causing a phase transition and increasing doxorubicin release [98].

## 5. Discussion

In situ phase transition is one of the most promising technologies in implantation today. The drug is delivered in a liquid form that is convenient for injection, with minimal risk of trauma, and without the use of surgical procedures. In addition, the polymers used for in situ formation are biodegradable, and such implants do not need to be removed once the drug has been released and the therapeutic effect has ended.

In situ implants are formed at the site of injection in response to pathological endogenous or exogenous factors, which may include temperature, the ionic composition of biological fluids, pH, tissue structure, irradiation, and enzyme composition. For tumors, depending on the type, size, and other factors, temperature and pH stimuli are considered the most selective because, unlike other pathologies, tumors are often characterized by acidosis and significant inflammation. At the same time, it should be noted that ion-selective polymers, according to the literature review, do not find their application in intratumoral in situ implantation.

The mechanisms of in situ implant formation depend on the nature of the active stimuli or their combinations.

Thermosensitive polymers are, by nature, amphiphilic polymers with hydrophilic and hydrophobic groups, potentially capable of micelle formation. A change in the solubility of the polymer in water is necessary for the phase transition to occur. Thermosensitive polymers are typically used at high concentrations in targeted delivery systems due to the need to overcome the critical micellar concentration for a temperature-dependent phase transition.

In contrast, phase-sensitive systems have the most stable stimulus potentiating the phase transition—solvent diffusion into the surrounding soft tissues or extraction with physiological fluids—indicating both the advantages of such systems (low dependence on external factors compared to, for example, thermosensitive matrices) and their disadvantages.

The mechanism of in situ phase transition common to all pH-sensitive polymers is swelling in an acidic environment and gel formation during the pH transition in the neutral or alkaline range by the formation of cross-links between chains of a swollen polymer with the formation of a three-dimensional gel structure. Carbomers, as well as salt forms of chitosan, work by such a mechanism. However, numerous studies have shown that the main introduction of acidic forms into the body does not agree with the desired profile of the osmotility and pH of drugs, so such systems rarely successfully overcome the stage of preclinical studies. Today, the pH-sensitive degradation of polymers is considered a more promising vector. A similar mechanism is used by Schiff bases based on chitosan and other polymers, such as ethylenediamine and dialdehyde pullulan, as well as zeolites [99,100].

Photosensitive polymers have three alternative in situ formation mechanisms [101]. The first is characterized by photothermal delivery systems, which collapse upon temperature elevation caused by IR irradiation, releasing the drug. Photodynamic systems contain photosensitizers that generate reactive oxygen species under light irradiation, causing cell death, which greatly contributes to the enhancement of chemotherapeutic efficacy and is often used for intratumoral implantation. Photoconversion systems incorporate structural blocks that convert IR to UV light. This approach allows known UV-sensitive monomers to be incorporated into the copolymer composition.

As a result of the analysis, a large number of polymers and related specialized techniques are currently known, which allow obtaining in situ implants with a high degree of directionality of action, which is an important factor for the intratumoral delivery of chemotherapeutic agents. The modern level of technologies, achieved through the harmonization of world research, allows the selection of the type of stimulating factor or combinations of various stimuli, depending on the pathogenesis of the tumor, its size, and other characteristics, thus making the therapy truly personalized.

It should be noted, however, that from a technological point of view, the development of systems with a specific stimulus involves various risks that must also be taken into account during the pharmaceutical development process.

Table 1 summarizes the characteristics of in situ systems for intratumoral implantation discussed above.

To address the emerging risks in the pharmaceutical development of certain stimulus-sensitive systems, various approaches can be applied, as discussed in the studies. For example, local tumor heating is used for targeting thermosensitive systems [78], and the correction of the explosive release of phase-sensitive systems can be performed by changing one of the matrix parameters—the hydrophobicity of the matrix-forming agent or solvent, loading of AFIs, etc. [92]. Due to the minimal invasiveness of in situ implantation inside the tumor, there is less trauma to the malignant neoplasm, and the risks of premature tumor collapse and cell dissemination in the body are reduced. At the same time, it should be noted that the majority of in situ implants considered in the reviewed publications are semi-solid structures, which does not allow them to resist the internal pressure of the tumor, which may lead to a decrease in the prolonged effect and “explosive” release of high doses of chemotherapeutic drugs, negating all the advantages of implants as sustained release forms.

The most promising are the multi-sensorial systems, which are directed to the complex of stimuli characterizing the tumor tissue. These in situ implants will be able to combine the positive properties of several systems at once and compensate for their disadvantages.

In 2023, the global pharmaceutical market will still have an extremely narrow range of in situ drugs. A review of drug registries from various countries did not reveal any in situ drugs for intratumoral implantation. One of the few approved drugs with in situ forming technology, Eligard^®^, is a subcutaneous implant. This in situ implant, which contains the active ingredient leuprorelin, is used for the palliative treatment of prostate cancer by reducing the body’s production of testosterone [117]. According to experts, the main disadvantage of the drug is the high critical release of leuprorelin—40% in the first 24 h [118,119]. Thus, the main part of modern, effective intratumoral implants of the latest generation is still at the stage of development or in vivo studies.

From 2004 to date, more than 15 clinical trials at major world universities (Oxford, Stanford, UCLA, University of Munich, etc.) have been dedicated to the study of ISS—ThermoDox^®^. ThermoDox^®^ (Celsion Corporation, USA) is a lysotherm-sensitive liposomal doxorubicin designed for targeted chemotherapy. Dipalmitoylphosphatidylcholine, a temperature-sensitive component of the liposome membrane, undergoes a phase transition with the release of the contents at 42 °C, which can be achieved clinically by local hyperthermia [120].

**Figure 1:**
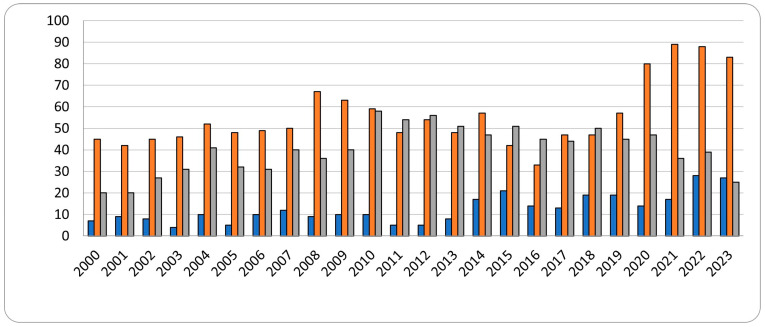
Number of publications per year (2000–2023) in the database of medical publications PubMed on request: “intratumoral injections” (orange column), “intratumoral implantation” (blue column), and “subcutaneous implantation” (grey column).

From the graph in Figure 1, we can see a trend of increasing investigator interest in “intratumoral injections” (orange column). This can be explained by the simplicity of the technology, the wide range of drugs used, the convenience of the research method, and the speed of obtaining results, as described in the subcutaneous implants section.

The least popular request in this case can be called “intratumoral implantation” (blue column) and “subcutaneous implantation” (grey column). In addition to the above-mentioned reasons, intratumoral implantation has high risks of the possibility of tumor damage due to the density of the implant, risks of damage to internal organs, high probability of experiencing failure due to the complexity of the procedure, as well as with the high expenditure of resources for research, which does not detract from the importance and great prospects for research on this topic. Nevertheless, despite the small number of experiments, we can observe a slight increase in interest in the data, which certainly gives a positive outlook for the future.

In conclusion, we can say that subcutaneous implants, like intratumoral implants, still show their potential, do not lose their popularity and relevance among scientists, and prove their promise for further development in the treatment of oncology and improving the level and quality of life of the population.

## 6. Conclusions

Intratumoral implantation is a modern method of delivering chemotherapeutic agents that are highly targeted and better tolerated by patients due to the absence of systemic effects. Implants formed directly after injection into the tumor (in situ systems) have significant advantages over classical solid forms for implantation into the malignancy. The phase transition stimulus underlying the function of such a targeting delivery system is a critical factor in the creation of an in situ system and should be chosen depending on the peculiarities of the pathogenesis of a particular tumor.

Further study of intratumoral implantation requires focusing on the behavior of neoplasms when their integrity is compromised, such as tumor rupture, increased intratumoral pressure, and increased tumor size or trauma. In addition, the study of the body’s immune response to implantation is an important aspect. Although implantation inside a tumor is technologically no different from a standard injection, the response of the body’s immune barriers may be unpredictable. With the additional study of such aspects of intratumoral implantation, it will be possible to incorporate this procedure into standard therapeutic practice for tumor diseases.

All existing problems of intratumoral in situ implants are due to insufficiently studied issues. The incidence of certain risks can only be reduced by conducting clinical trials and proving the safety and efficacy of intratumoral implantation, as has been repeatedly demonstrated in animal models.

## Figures and Tables

**Table 1:** Generalized characteristics of different in situ systems for intratumoral implantation.

Type of In Situ Formation Stimulus	Polymer Examples	Positive Characteristics	Negative Characteristics
Temperature	Poloxamers [8,65], chitosan derivatives [102], PLGA-PEG, PNIPAAm [103], xyloglucan [104]	The most studied stimulus Commercially available polymer—poloxamer 407 Large range of polymers	Low selectivity High polymer concentrations Dependence of phase transition on pH for some polymers Low stability of the stimulus for systems during storage
pH	Chitosan salts [105,106,107], carbomers [108,109], zeolites [110,111,112]	Selectivity to local pathological acidosis	No commercially available polymers Non-physiologic pH at injection (hyperosmolarity) Low bioadhesion characteristics Difficulty in controlling release parameters
Phase inversion	Polycaprolactone (PCL) PLGA [90,113], shellac [114,115]	High stimulus stability Commercially available polymers	Explosive release of APIs Solvent non-differentiation
UV-light	Polyethylene glycol diacrylate (PEGDA), gelatin methacrylate (GelMA), methacrylated hyaluronic acid (MeHA) [116], polyethylene glycol-polynitrobenzyl glutamate [87], *N*-phenylglycine-polyethylene glycol [91], etc.	High stimulus stability Easy medical manipulation for activation	Directed polymer synthesis Suitable for superficial tumors only
Other	Diphenylalanine dipeptide derivatives [92], PDLLA-PEG-PDLLA [94], etc.	High selectivity	Small pool of experimental data The difficulty of targeting Directed polymer synthesis

## Data Availability

The data presented in this study are openly available in article.
